# Differential Effects of Early Weaning for HIV-Free Survival of Children Born to HIV-Infected Mothers by Severity of Maternal Disease

**DOI:** 10.1371/journal.pone.0006059

**Published:** 2009-06-26

**Authors:** Louise Kuhn, Grace M. Aldrovandi, Moses Sinkala, Chipepo Kankasa, Katherine Semrau, Prisca Kasonde, Mwiya Mwiya, Wei-Yann Tsai, Donald M. Thea, for the Zambia Exclusive Breastfeeding Study (ZEBS)

**Affiliations:** 1 Gertrude H. Sergievsky Center, Department of Epidemiology, Mailman School of Public Health, Columbia University, New York, New York, United States of America; 2 Department of Pediatrics, Children's Hospital Los Angeles, University of Southern California, Los Angeles, California, United States of America; 3 Lusaka District Health Management Team, Lusaka, Zambia; 4 University Teaching Hospital, University of Zambia, Lusaka, Zambia; 5 Center for International Health and Development, Boston University School of Public Health, Boston, Massachusetts, United States of America; 6 Department of Biostatistics, Mailman School of Public Health, Columbia University, New York, New York, United States of America; Medical Research Council South Africa, South Africa

## Abstract

**Background:**

We previously reported no benefit of early weaning for HIV-free survival of children born to HIV-infected mothers in intent-to-treat analyses. Since early weaning was poorly accepted, we conducted a secondary analysis to investigate whether beneficial effects may have been hidden.

**Methods:**

958 HIV-infected women in Lusaka, Zambia, were randomized to abrupt weaning at 4 months (intervention) or to continued breastfeeding (control). Children were followed to 24 months with regular HIV PCR tests and examinations to determine HIV infection or death. Detailed behavioral data were collected on when all breastfeeding ended. Most participants were recruited before antiretroviral treatment (ART) became available. We compared outcomes among mother-child pairs who weaned earlier or later than intended by study design adjusting for potential confounders.

**Results:**

Of infants alive, uninfected and still breastfeeding at 4 months in the intervention group, 16.1% who weaned as instructed acquired HIV or died by 24 months compared to 16.0% who did not comply (p = 0.98). Children of women with less severe disease during pregnancy (not eligible for ART) had worse outcomes if their mothers weaned as instructed (RH = 2.60 95% CI: 1.06–6.36) compared to those who continued breastfeeding. Conversely, children of mothers with more severe disease (eligible for ART but did not receive it) who weaned early had better outcomes (p-value interaction = 0.002). In the control group, weaning before 15 months was associated with 3.94-fold (95% CI: 1.65–9.39) increase in HIV infection or death among infants of mothers with less severe disease.

**Conclusion:**

Incomplete adherence did not mask a benefit of early weaning. On the contrary, for women with less severe disease, early weaning was harmful and continued breastfeeding resulted in better outcomes. For women with more advanced disease, ART should be given during pregnancy for maternal health and to reduce transmission, including through breastfeeding.

**Trial Registration:**

Clinical trials.gov NCT00310726

## Introduction

Global public health recommendations advise exclusive breastfeeding for the first six months of life followed by introduction of nutritionally-adequate complementary foods while breastfeeding continues to two years or beyond [1]. For infants of HIV-infected mothers, however, mathematical models have estimated that there may be benefits of shortening the usual duration of breastfeeding [2], [3] and several studies have observed ongoing risks of HIV transmission with continued breast milk exposure regardless of the child's age [4]–[7]. These findings prompted several authors to suggest that early weaning may be advisable for HIV-infected women [4]–[7]. None of these studies has taken into account the competing risks of uninfected child mortality as a result of early termination of breastfeeding.

We conducted a randomized clinical trial in Lusaka, Zambia, of a counseling program to encourage early weaning at 4 months of age to determine the effects of this intervention on HIV-free survival of infants born to HIV-infected mothers [8]. Although we expected that early weaning would be beneficial, there were no empirical data to support this belief at the time we started the trial. In final intent-to-treat analyses, no benefit of early weaning for HIV-free survival was observed [9]. Since early weaning was poorly accepted by the study population despite intensive counseling and support to encourage it, some have argued that incomplete adherence may have masked a true benefit of the intervention [10]. Here we present secondary analyses to investigate the consequences of incomplete adherence for inferences about the effects of early weaning, comparing outcomes among mother-child pairs in the intervention and control groups who stopped breastfeeding earlier or later than intended by the study design.

## Methods

### Study design

The study was an un-blinded, randomized trial of a behavioral intervention among HIV-infected women to encourage exclusive breastfeeding with abrupt cessation of breastfeeding at 4 months compared to standard practice [8]. In brief, HIV-infected women were recruited from two antenatal clinics in Lusaka, Zambia and received single-dose nevirapine prophylaxis [11], [12]. Between May 2001 and September 2004, 1435 pregnant, HIV-seropositive women who intended to breastfeed were recruited. Women still breastfeeding at 1 month post-partum (n = 958) were randomized to one of two groups. Randomization assignment was prepared by the study statistician and was accessed through a computer program. In the intervention group, women were encouraged to breastfeed exclusively to 4 months and then to stop breastfeeding abruptly. Preparation for early weaning began around two months with education, counseling and encouragement to express breast milk and practice cup feeding prior to weaning. A three-month supply of infant formula and a fortified weaning cereal was provided and training and education to make replacement feeding as safe as possible was done. In the control group, women were encouraged to exclusively breastfeed to 6 months, gradually introduce complementary foods (not provided) and continue to breastfeed for a duration of their own informed choice. All women provided written informed consent. The study was approved by Human Subjects Committees at the institutions of all the authors in the U.S. and in Zambia. The protocol for this trial and the supporting CONSORT checklist are available as supporting information; see Checklist S1 and Protocol S1.

### Study procedures

Maternal blood was drawn at enrollment and was tested for CD4 and CD8 counts (FACSCount, BD Immunocytometry Systems, San Jose, CA), hemoglobin (Hemocue® system, Lake Forest, CA) and viral load (Roche Amplicor® 1.5, Roche, Branchburg, NJ). Most women did not receive antiretroviral therapy (ART) for their own health as HIV treatment only became available in the public sector after May 2004 [13]. However, because data on CD4 count and clinical staging were collected at enrollment, we classified women retrospectively as having severe disease requiring ART if their CD4 count was <200 cells/µL or if their CD4 count was 200–349 cells/µL and they had WHO stage III or greater clinical disease. If women did not meet these criteria they were classified as having less severe disease. Cotrimoxazole was given to women with CD4 counts <200 after November 2003 [14]. Sociodemographic and clinical data were collected at enrollment and obstetric/neonatal data after delivery. Infant heelstick blood samples were collected onto filter paper on the day of birth, at 1 week, and at 1, 2, 3, 4, 4 ½, 5, 6, 9, 12, 15, 18, 21, and 24 months of age. Samples were tested in batches for HIV-1 DNA by PCR [15]. All positives were confirmed ≥2 samples if available, and, if not, the same sample was re-tested to confirm. To rule out false negative test results due to inadequate samples, amplification of the beta-globin gene was performed. Growth monitoring was done monthly and children from either group with evidence of failure-to-thrive were provided with nutrition supplements. Cotrimoxazole was given to all infants (infected and uninfected) between 6 weeks and 12 months. Home visit teams tracked participants who did not return for appointments. Information about child deaths was sought from hospital and clinic records and interviews with caretakers and health care personnel. Circumstances of all deaths were reviewed to identify causes of death. A standardized questionnaire was administered at every study visit by a member of the study team who was not conducting the feeding counseling to determine feeding behaviors in the past 24 hours, past week and since the last visit.

### Statistical methods

The analysis was restricted to 661 mother-child pairs who survived uninfected and still breastfeeding to 4 months. Since practices were intentionally the same in the two groups before this time, focus on the post-4 month time period provides the clearest investigation of the effects of actual behaviors on outcomes. Breastfeeding duration was determined based on the exact age that breastfeeding was first reported to have completely stopped. Children who died were assumed to have been breastfed up to their death date unless reports indicated that it ceased prior to the illness preceding the child's death. The primary outcome was HIV infection or death which was examined using Kaplan-Meier life-table methods for univariate and Cox Proportional Hazards models for multivariate analyses [16]. For HIV infection, the midpoint between the last negative and first positive PCR test was imputed as the event time, for deaths the actual age of death was used.

First, we examined the outcome (HIV infection or death) only in the intervention group stratifying by a fixed covariate to compare those who adhered to the intervention (defined as stopping breastfeeding by the completion of 4 months) vs. those who did not adhere (i.e. continued breastfeeding for 5 or more months). Other variables thought to be possible confounders or effect modifiers (maternal CD4 count, viral load, hemoglobin, body mass index, eligibility for ART, age, parity, RPR status, disclosure, education, marital status, domestic electricity, water source, cooking method, household crowding, other children in the home, past history of child deaths, employment, food security, compliance with nevirapine prophylaxis and child sex and birth weight) were entered into a Cox Proportional Hazards model on their own, with each other, and as possible effect modifiers of feeding practice. Variables that were significantly associated with the outcome or which changed the magnitude of the association between feeding practice and the outcome by more than 10% were retained in final multivariable models. Follow-up time was censored at the time women initiated ART.

Second, we restricted analyses to the control group and examined associations between the outcome (HIV infection or death) and breastfeeding cessation (weaning) as a time-dependent variable over the full duration of follow-up (24 months). Investigation of confounding and effect modification was done as in the intervention group.

Since our results identified severity of maternal disease as a major effect modifier, we conducted further analyses to identify an age when weaning would be “safe” We defined “safe” as the age when the weaning hazard ratio (HR) was less than or equal to 1. Combining data from both groups and restricted to those with less severe disease, we ran a Cox Proportional hazard model with one time-dependent covariate equal to 1 if weaning occurred <365 days (0 otherwise – var 1), and a second time-dependent covariate equal to 1 if weaning occurred 365–730 days (0 otherwise – var 2), and a multiplicative interaction term between the time-dependent covariate for weaning 365–730 days and child age of weaning (var 3). It was necessary to have two time-dependent terms for weaning since the HR associated with weaning increased with child age up to 12 months. To determine the exact child age when the HR reached 1 (i.e. no longer increased the risk of HIV infection or death), we took the ratio of the Cox model β coefficients for var 2 and var 3 i.e. β2/β3. This formula can be derived from the Cox model equation since e^β^ = HR or β = 0 when HR = 1 [16]. Utilizing this optimal age for weaning, we followed the same approach to identify a CD4 count threshold when weaning would be “safe.” Combining results from both groups and including women with both less and more severe disease, we ran a Cox model with CD4 count as a continuous variable (var 4), a time-dependent covariate equal to 1 if weaning occurred before the optimally selected age (0 otherwise – var 5), and a multiplicative interaction term between weaning and CD4 count (var 6). To estimate the exact CD4 count when the weaning HR crossed over from being beneficial (i.e. reducing the risk of HIV infections or deaths) to being harmful (i.e. increasing the risk of HIV infections or death), we took the ratio of β5/β6.

To describe differences between groups, categorical characteristics were tested using chi-squared tests, normally-distributed continuous variables using t-tests, non-normal continuous variables using Wilcoxon tests and time to event variables using log-rank tests. To compare breastfeeding duration between groups, we treated breastfeeding as a time-to-event variable with weaning as the event and censoring pairs who were lost or died while still breastfeeding. Kaplan-Meier life-table methods were used for univariate and Cox Proportional Hazards models for multivariate analyses [16]. All analyses were done using SAS version 9.2 (Cary, NC).

## Results

### Study population

Of 958 mother-child pairs randomized, 328/481 (68.2%) in the intervention and 333/477 (69.8%) in the control group were alive, HIV uninfected (HIV negative PCR at ≥4 months) and still breastfeeding at 4 months. In the intervention group of those still breastfeeding at 4 months, 60.9% stopped breastfeeding by the completion of 4 months (as dictated by the study protocol) vs. 2.1% among the controls. The median duration of breastfeeding was 4.4 months (inter-quartile range [IQR] 4.2–15 months) in the intervention and 16 months (IQR 12–19 months) in the control group (p<0.001).

### Effects of adherence in the intervention group

There was no significant difference in the rate at which children of mothers who adhered to the intervention and weaned by the end of 4 months acquired HIV infection or died by 24 months (16.1%) compared to those who were non-adherent and continued breastfeeding (16.0% p = 0.98). If person-time was censored at the time of ART initiation, the rates of HIV infection or death by 24 months were 16.2% among those who weaned at 4 months vs. 14.2% among those who continued breastfeeding for longer (p = 0.64). There were some differences between mother-child pairs who adhered to the intervention counseling (Table 1) but median CD4 counts and viral loads were not significantly different between those mothers who adhered (348 cells/ml and 33,360 copies/ml) vs. those who did not (373 cells/ml and 27,972 copies/ml, respectively).

**Table 1:** Predictors of adherence (i.e. weaning at 4 months) among 328 mother-child pairs randomized to the intervention group surviving HIV-free and still breastfeeding at 4 months.

Characteristic	Total N	N (%) stopped BF by end of 4 mo	p-value
***Maternal factors:***			
CD4 count			
<200	60	40 (66.7)	
200–349	100	65 (65.0)	
≥350 cells/mm^3^	168	98 (58.3)	>0.10
Plasma viral load			
≤9,999	99	61 (61.6)	
10,000–99,999	157	88 (56.1)	
≥100,000 copies/ml	71	54 (76.1)	0.02
Hemoglobin			
<10	79	53 (67.1)	
≥10 g/dL	246	149 (60.6)	>0.10
Classification of severity of disease*			
Less severe	229	134 (58.5)	
More severe	99	69 (69.7)	0.06
Body mass index 1 month post-partum			
<18.5	48	34 (70.8)	
≥18.5	280	169 (60.4)	>0.10
Age (years)			
<20	19	14 (73.7)	
20–24	115	62 (53.9)	
25–29	101	67 (66.3)	
≥30	93	60 (64.5)	>0.10
Parity			
First child	39	23 (59.0)	
2^nd^–3^rd^	156	92 (59.0)	
≥Fourth	133	88 (66.2)	>0.10
RPR status			
Positive	56	33 (58.9)	
Negative	252	163 (64.7)	>0.10
***Social factors:***			
Disclosed HIV status to partner	182	113 (62.1)	
Not disclosed	146	90 (61.6)	>0.10
Marital status			
Married	284	175 (61.2)	
Single	29	17 (58.6)	
Widowed/divorced/separated	15	11 (73.3)	>0.10
Education			
No school or <8 years of school	188	107 (52.7)	
Some or complete high school (≥8 y)	140	96 (68.6)	0.03
Domestic water source			
Within dwelling or on property	51	44 (86.3)	
Communal	277	159 (57.4)	<0.01
Electricity in the home			
Yes	132	87 (65.9)	
No	196	116 (59.2)	>0.10
Cooking facilities			
Stove/hotplate	117	76 (65.0)	
Charcoal/wood	211	127 (60.2)	>0.10
Full-/part-time or informal sector job	98	69 (69.7)	
Unemployed	229	134 (58.5)	0.06
***Child characteristics:***			
Infant sex			
Male	175	104 (59.4)	
Female	153	99 (64.7)	>0.10
Birth weight			
<2,500 grams	28	19 (67.9)	
≥2,500 grams	294	182 (61.9)	>0.10

* Less severe disease was defined as CD4 counts >350 cells/µL during pregnancy or if between 200 and 349 cells/µL had to be classified as WHO stage II or less; more severe disease was defined as CD4 count <200 cells/µL or if CD4 count 200–349 cells/µL had to be classified as WHO stage III or greater.

**Figure 1:**
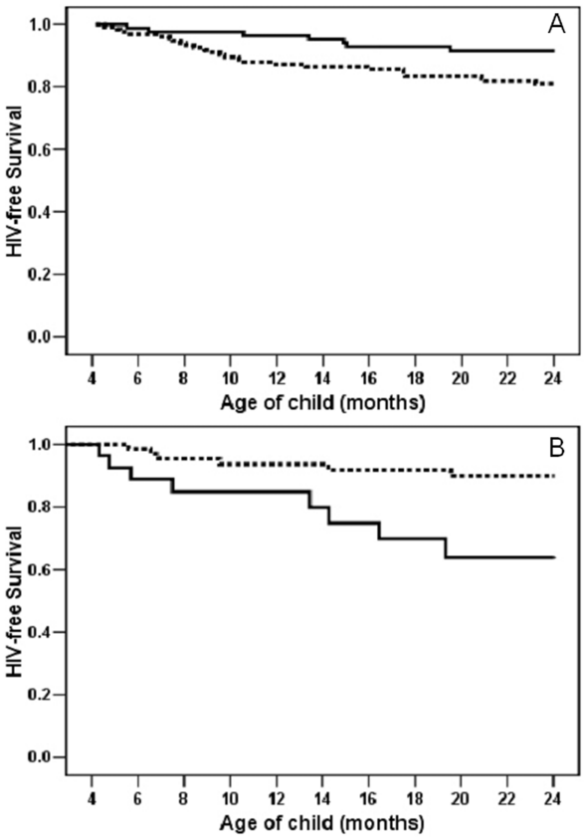
Panel A: HIV-free survival among 229 children in the intervention group whose mothers had less severe disease and would not have been eligible for antiretroviral therapy during pregnancy stratified by whether they adhered to the intervention (weaned by the completion of 4 months) (dashed line) or whether they continued breastfeeding after 4 months (solid line) (p = 0.035). Panel B: HIV-free survival among 97 children in the intervention group whose mothers had advanced disease and would have been eligible for antiretroviral therapy during pregnancy but did not receive it stratified by whether they adhered (weaned by the completion of 4 months) (dashed line) or whether they continued breastfeeding after 4 months (solid line) (p = 0.006).

There was significant effect modification by the severity of maternal disease. Children of women with less severe disease during pregnancy had significantly worse outcomes if their mothers were adherent and weaned early compared to infants whose mothers were non-adherent and continued breastfeeding (RH = 2.40 95% CI: 1.04–5.54). In contrast, children born to women with advanced HIV disease had significantly better outcomes if their mothers were adherent (Figure 1). The interaction was significant (p = 0.001) and early weaning remained associated with a more than 2-fold increase in the risk of HIV infection or death (RH = 2.60 95% CI: 1.06–6.36, p = 0.036) among infants born to mothers with less severe disease even after adjusting for maternal hemoglobin, body mass index, parity, and birth weight. No other factors met criteria for inclusion in final multivariable models.

The differential effects of early weaning by the severity of maternal disease were explained by low rates of HIV transmission and elevated uninfected child mortality due to weaning among women with less severe disease. Among women with less advanced disease, HIV transmission rates were low regardless of whether or not early weaning was practiced (5.0%) or not (3.9%) but uninfected child mortality rates were significantly higher among children whose mothers adhered to the intervention (14.8%) compared to those who did not (4.7%). As a result there was net harm associated with adherence with the intervention among infants born to mothers with less severe disease. Among women with advanced disease, HIV transmission rates were significantly lower among those who weaned early (5.5%) compared to those who continued to breastfeed (25.6%) and, in this group, uninfected child mortality was not affected by weaning practice, resulting in a net benefit associated with adherence with the intervention (Table 2).

**Table 2:** In the intervention group, effects of adherence (weaning at 4 months) on HIV transmission and uninfected child mortality stratified by the severity of maternal HIV disease during pregnancy.

Child outcome:	Less severe maternal disease*					More severe maternal disease*				
	Mother not eligible for antiretroviral therapy					Mother eligible for antiretroviral therapy				
	N in group	N with outcome	Prob. of outcome	Hazard ratio (95% CI)	Log-rank p-value	N in group	N with outcome	Prob. of outcome	Hazard ratio (95% CI)	Log-rank p-value
HIV infection or death										
Adherent**	134	25	0.190	2.40 (1.04–5.54)	0.035	68	6	0.101	0.25 (0.09–0.72)	0.006
Not adherent	95	7	0.085	Ref.		29	8	0.360	Ref	
HIV infection										
Adherent**	134	6	0.047	1.38 (0.34–5.51)	0.68	68	3	0.048	0.21 (0.05–0.87)	0.01
Not adherent	95	3	0.038	Ref.		29	5	0.208	Ref.	
Uninfected death										
Adherent**	134	19	0.148	3.23 (1.10–9.50)	0.024	68	3	0.047	0.36 (0.07–1.77)	0.19
Not adherent	95	4	0.047	Ref.		29	3	0.126	Ref.	

* Less severe disease was defined as CD4 counts >350 cells/µL during pregnancy or if between 200 and 349 cells/µL had to be classified as WHO stage II or less; more severe disease was defined as CD4 count <200 cells/µL or if CD4 count 200–349 cells/µL had to be classified as WHO stage III or greater.

** Adherent was defined as stopping all breastfeeding by the completion of 4 months.

### Effects of spill-over in the control group

Breastfeeding duration in the control group (median 16 months) was shorter than expected based on reported behaviors during the prior pregnancy (median 18 months). Weaning before 4 months was rare (2.1%) but cessation before 12 months (21.4%) was more frequent than reported for the previous pregnancy (13.5%). Factors associated with the duration of breastfeeding in the control group are shown in Table 3. More education and the availability of water and electricity were associated with shorter breastfeeding.

**Table 3:** Predictors of breast feeding duration among 333 mother-child pairs randomized to the control group surviving HIV-free and still breastfeeding at 4 months.

Characteristic	Total N	Median duration BF (months)	p-value
***Maternal factors:***			
CD4 count			
<200	64	15.0	
200–349	95	16.0	
≥350 cells/mm^3^	174	17.0	>0.10
Plasma viral load			
≤9,999	99	16.0	
10,000–99,999	155	16.0	
≥100,000 copies/ml	78	17.0	>0.10
Hemoglobin			
<10	88	16.0	
≥10 g/dL	238	16.0	>0.10
Classification of severity of disease*			
Less severe	228	17.0	
More severe	105	15.2	>0.10
Body mass index 1 month post-partum			
<18.5	44	17.0	
≥18.5	288	16.0	>0.10
Age (years)			
<20	28	15.0	
20–24	111	16.0	
25–29	107	17.0	
≥30	87	16.0	>0.10
Parity			
First child	43	15.3	
2^nd^–3^rd^	151	16.0	
≥Fourth	139	17.0	>0.10
RPR status			
Positive	55	18.0	
Negative	255	16.0	>0.10
***Social factors:***			
Disclosed HIV status to partner	198	16.0	
Not disclosed	135	16.5	>0.10
Marital status			
Married	281	16.0	
Single	33	14.0	
Widowed/divorced/separated	19	17.0	>0.10
Education			
No school or <8 years of school	177	17.3	
Some or complete high school (≥8 y)	156	15.0	0.004
Domestic water source			
Within dwelling or on property	51	15.0	
Communal	282	16.7	0.007
Electricity in the home			
Yes	134	15.0	
No	199	17.0	0.002
Cooking facilities			
Stove/hotplate	117	15.0	
Charcoal/wood	215	17.0	0.0002
Full-/part-time or informal sector job	110	16.0	
Unemployed	223	16.0	>0.10
***Child characteristics:***			
Infant sex			
Male	174	17.0	
Female	158	16.0	>0.10
Birth weight			
<2,500 grams	34	18.0	
≥2,500 grams	293	16.0	>0.10

* Less severe disease was defined as CD4 counts >350 cells/µL during pregnancy or if between 200 and 349 cells/µL had to be classified as WHO stage II or less; more severe disease was defined as CD4 count <200 cells/µL or if CD4 count 200–349 cells/µL had to be classified as WHO stage III or greater.

Consistent with the results in the intervention group, a statistically significant interaction (p = 0.01) between breastfeeding cessation and severity of maternal disease was detected in the control group. Among infants of mothers with less severe disease, stopping breastfeeding before 24 months was associated with >3-fold increased risk of HIV infection or death (RH = 3.41 95% CI: 1.52–7.65, p = 0.003) whereas among infants of mothers with more advanced disease, weaning was not associated with the outcome adjusting for maternal viral load and child birth weight. No other factors met criteria for inclusion in final multivariable models.

### Thresholds for safer weaning

Among infants born to HIV-infected mothers with less advanced disease in either randomized group, breastfeeding remained protective until 15 months age; thereafter there was no longer any excess risk of the combined outcome of HIV infection or death. Effects of weaning before 15 months were consistent between the intervention and control groups and remained after adjusting for potential confounders. In the intervention group, weaning before 15 completed months was associated with a 3.34–fold increase in the risk of HIV infection or death (95% CI: 1.16–9.59) and in the control group a 3.94-fold increase (95% CI: 1.65–9.39) among those with less severe disease and after adjusting for confounders (Table 4).

**Table 4:** Effects of weaning before 15 months on the combined outcome of HIV transmission or uninfected child mortality stratified by the severity of maternal HIV disease in the intervention and control groups separately.

Maternal disease status during pregnancy*	*Intervention Group*		*Control Group*	
	Hazard ratio (95% CI)	p-value	Hazard ratio (95% CI)	p-value
***Less severe***				
Stop BF ≤15 months	3.34 (1.16–9.59)	0.025	3.94 (1.65–9.39)	0.002
***More severe***				
Stop BF ≤15 months	0.24 (0.07–0.85)	0.027	0.72 (0.23–2.29)	
***p-value interaction***		0.001		0.01

* Less severe disease was defined as CD4 counts >350 cells/µL during pregnancy or if between 200 and 349 cells/µL had to be classified as WHO stage II or less; more severe disease was defined as CD4 count <200 cells/µL or if CD4 count 200–349 cells/µL had to be classified as WHO stage III or greater.

† Hazard ratios are from Cox Proportional Hazards models treating breast feeding cessation before 15 months as a time-dependent covariate adjusting for maternal viral load, hemoglobin, body mass index, parity and child birth weight. Hazard ratios >1 indicate that weaning increases the risk of the combined outcome of HIV infection or death. Hazard ratios <1 indicate that weaning decreases the risk of the outcome.

**Figure 2:**
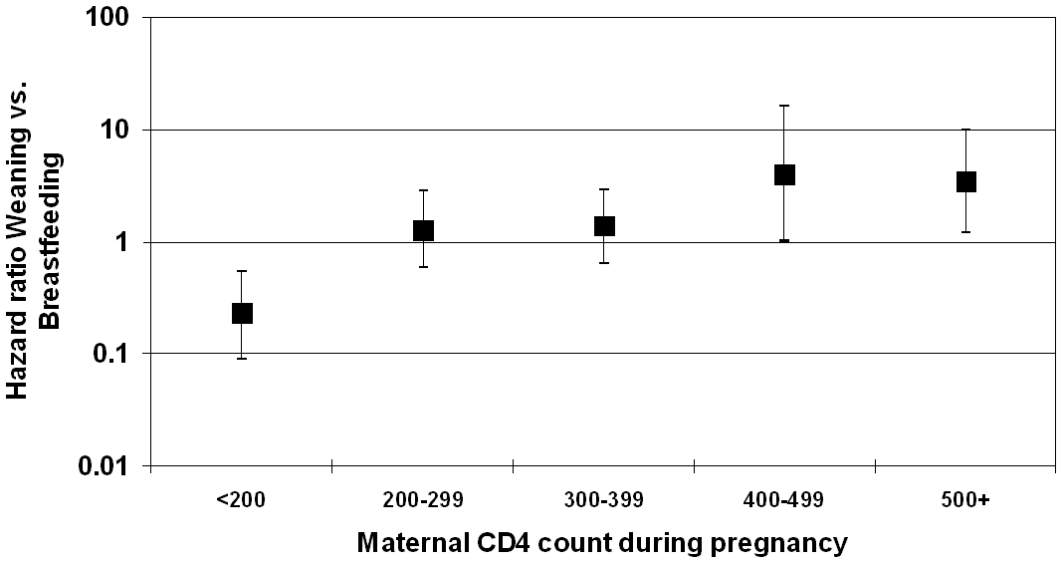
Relative hazards of HIV infection or death by weaning before 15 completed months stratified by maternal CD4 count during pregnancy. Hazard ratios greater than 1 indicate that weaning increases the risk of HIV/death. Hazard ratios less than 1 indicate that weaning decreases the risk of HIV/death.

Using as a reference breastfeeding to 15 months, weaning before 15 months was associated with reduced risk of HIV infection or death when maternal CD4 counts were low and increased risk of HIV infection or death when maternal CD4 counts were high (Figure 2). The threshold CD4 count identified by the data was 306 cells/µL (95% CI: 220–394). In other words, weaning was protective if women had CD4 counts below this cut-point, and was harmful if women had higher CD4 counts.

## Discussion

All clinical trials with behavioral interventions are vulnerable to misleading results depending on the degree of adherence. Poor adherence may dilute the outcomes of a beneficial intervention leading to the erroneous conclusion of no benefit. However, if an intervention is harmful, then poor adherence may lead to the erroneous conclusion that an intervention is safe when it is not. The primary outcome of our trial was a combination of HIV infection or uninfected child death and early weaning affects each of these outcomes differently. The analysis we present here demonstrates that, after taking into account the dilution introduced by poor adherence, gains achieved in transmission reduction due to early weaning were offset by increases in mortality in the group overall. Hence the original conclusion of no benefit of early weaning is correct if no distinction is made among mothers at different stages of HIV progression.

Investigation into possible confounding led us to identify an interaction between severity of maternal HIV disease during pregnancy and effects of early weaning. This interaction has profound implications for infant feeding policies for HIV-infected women in low resource settings. If infants were born to mothers with advanced HIV disease, i.e. symptomatic and low CD4 counts, then early weaning resulted in fewer HIV infections or deaths. If infants were born to mothers with less advanced HIV disease, i.e. asymptomatic and high CD4 counts, then early weaning resulted in more adverse clinical outcomes. Since women with advanced disease should receive ART routinely as part of HIV treatment services [17], we anticipate that post-natal transmission should also be reduced. This has been shown in some demonstration projects [18]–[20]. Therefore, if access to ART is in place, there is little justification for shortening the duration of breastfeeding among mothers with advanced disease. For women who do not yet need ART according to usual guidelines, our data indicate that risks associated with transmission are not large enough to outweigh the substantial increases in uninfected child mortality that result from prematurely truncating the usual duration of breastfeeding. For infants of mothers with less severe disease, weaning at 15 months or earlier is associated with a more than 3-fold increase in HIV infection or death. Even taking into account continuing risks of HIV transmission, HIV-free survival is better if breastfeeding continues for at least 16 months.

There are new data to support proof of concept that antiretroviral drugs given to the infant during breastfeeding can reduce postnatal transmission [21], [22]. Unfortunately, the studies investigating this have tested only short interventions [21], [22] premised on the assumption that infants of HIV-infected women could be safely weaned early. Studies are needed to evaluate interventions that extend over a full ordinary duration of breastfeeding.

A limitation of our study was that adherence with the intervention was incomplete. An advantage of intent-to-treat analysis of randomized controlled trials is that confounders associated with the practice of interest are less likely to explain associations. However, substantial lack of adherence with an intervention may bias associations towards the null, making intent-to-treat results difficult to interpret. We had not originally anticipated that early weaning would be so poorly accepted in our study population. The secondary analysis we present here is, like all analyses of epidemiological data, vulnerable to confounding and reverse causation. To address reverse causation, we reviewed the circumstances of all deaths, and weaning was only classified to have occurred if breastfeeding had ceased before the illness that preceded the child's death, and not as a result of that illness. We acknowledge that this cannot be foolproof but our approach avoids one of the methodological problems that has been previously identified in the field of breastfeeding research [23]. We also examined a large number of confounders, including several known to be strong predictors of HIV transmission, specifically maternal CD4 count and viral load, and those known to be strong predictors of child mortality, namely birth weight and socioeconomic standing. None of these factors explained away the weaning associations. However, it is possible that other unmeasured confounders may account for our associations. The associations between weaning and adverse outcomes were consistent across the intervention and control groups. This makes it highly unlikely that social desirability tendencies or reporting biases (which would operate in different directions in the two groups) would explain the findings. Moreover, the benefits of continued breastfeeding in the women without advanced HIV disease are similar to those reported in non HIV infected populations [24].

### Conclusions

Current WHO guidelines encourage weaning to occur only once affordable, feasible, acceptable, sustainable, and safe (AFASS) alternatives to breast milk are available [25]. If guidelines are followed, HIV-infected women would only have initiated breastfeeding if AFASS criteria were not met during pregnancy. Since socioeconomic circumstances are unlikely to change between pregnancy and 6 months, it is unclear what this recommendation means for the appropriate age of weaning. Using HIV-free survival as the outcome, our data demonstrate that women from urban, resource-limited settings, who have high CD4 counts, place their infants at significantly increased risk if breastfeeding is stopped at any time before 16 months. There is a net benefit of continued breastfeeding for HIV-free survival of infants when maternal CD4 counts exceed about 300 cells/ul. For women with lower CD4 counts, initiating ART as expeditiously as possible during pregnancy should be an urgent priority, for their own health and to reduce transmission to the child.

## Supporting Information

Checklist S1 CONSORT Checklist
(0.05 MB DOC)

Protocol S1 Trial protocol
(0.13 MB DOC)
