# Supplementary material for: Differential Effects of Early Weaning for HIV-Free Survival of Children Born to HIV-Infected Mothers by Severity of Maternal Disease
Source: PLoS One. 2009 Jun 26;4(6):e6059. doi: 10.1371/journal.pone.0006059 (PMC2698120; doi:10.1371/journal.pone.0006059)
Supplement: Protocol S1 — Trial protocol (0.13 MB DOC) [file pone.0006059.s002.doc]

Protocol

##### Short Exclusive Breast Feeding to Reduce Postnatal Transmission of HIV Through Breastmilk

**A- Study Purpose and Rationale**

It is well established that infants breast fed by their HIV-infected mothers are at risk of acquiring HIV infection through breast milk. However, in low resource settings where the HIV epidemic now predominates, breast feeding cannot simply be replaced by breast milk substitutes. Alternatives to breast milk are often unavailable or unaffordable and avoidance of breast feeding substantially increases the risk and severity of malnutrition and other diseases such as diarrhea and pneumonia. Recent findings suggest a reduced risk of HIV transmission when breast feeding is exclusive (infants only receive breast milk) compared to the more common practice of mixed feeding (infants are given other liquids and foods in addition to breast feedings). The type of breast feeding also affects non-HIV morbidity and mortality as exclusive breast feeding provides greater protection than mixed feeding.

This study aims to test the safety and efficacy of short duration exclusive breast feeding to minimize the risks of HIV transmission without increasing risks of non-HIV infant mortality. The primary objectives of the study are (1) to compare HIV transmission rates in the first 4 months of life associated with exclusive breast feeding compared to nonexclusive breast feeding; (2) to test if there is an overall benefit of early cessation of breast feeding on HIV-infection-free survival; (3) to estimate the magnitude of reduction in HIV transmission attributable to cessation of breast feeding at 4 months and (4) to estimate the magnitude of the increase in non-HIV-related under-2-year mortality attributable to cessation of breast feeding at 4 months. Secondary objectives are to describe acute and chronic effects of abrupt weaning on child morbidity. The study proposes to test an inexpensive and potentially sustainable public health intervention to reduce HIV transmission through breast feeding while preserving benefits of breast feeding for other aspects of child health in a very low resource setting.

**B- Study Design and Statistical Analysis**

The study is designed as a randomized un-blinded preventive trial (prospective follow-up study) with specific observational components specified *a priori*. All women who enroll for prenatal care services at two primary health clinics in Lusaka, Zambia (George Clinic or Chawama Clinic) will be offered testing for HIV-antibody. HIV testing will be voluntary, with signed, informed consent and pre- and post-test counseling. Women will be counseled by HIV counselors trained in Zambia following local counseling guidelines. The two-dose nevirapine therapy will be available for all HIV+ women to reduce mother-to-child transmission regardless of participation in the research study.

As part of the post-test counseling, all women who test HIV+ will receive information regarding the risks and benefits of breast feeding and will be asked to make an informed choice regarding infant feeding. HIV+ women who indicate their decision to breast feed will be eligible for enrollment into the study. The requirements of the study will be explained and a second, signed, informed consent will be obtained. All women who agree to participate in the study will be counseled to breast feed exclusively to 4 months. Half of these women (regardless of their actual feeding practice) will be randomized to a counseling program that will encourage abrupt cessation of breast feeding at 4 months and transition to full replacement feeding, and half will be randomized to a counseling program consistent with standard practice (gradual introduction of weaning foods after 4 months).

Schematic outline of the study design

| Prenatal | All HIV+ women: Offered nevirapine & post-test counseling to include choice of infant feeding | | | | | | | | |  |
| --- | --- | --- | --- | --- | --- | --- | --- | --- | --- | --- |
|  |  | |  | | | | |  | |  |
|  |  | | Those who indicate their intention to breast feed eligible for enrollment | | | | |  | |  |
|  |  | |  | | | | |  | |  |
|  |  | | All enrolled women counseled to  exclusively breast feed to 4 months | | | | |  | |  |
|  |  | |  | |  |  | |  | |  |
| 4 months |  | |  | | Randomization |  | |  | |  |
|  | |  | |  | | |  | |  | |
|  | | Abrupt cessation of all  breast feeding | |  | | | Continued breast feeding | |  | |
|  | |  | |  | | |  | |  | |
| 24 months | | HIV infections and deaths | |  | | | HIV infections and deaths | |  | |

At the one month visit post-delivery, a sealed envelope with a computer-generated random assignment will be opened and women will be informed of their random assignment. For women in the abrupt weaning group, counselors will work out with each individual mother a suitable plan to facilitate abrupt weaning. Food supplements will be given to all women in abrupt weaning group and will be made available for all others who need it.

Women and their children will be followed prospectively with regular study visits for two years to determine the HIV status of the child and to estimate under-2-year mortality.

The primary statistical analy­ses will compare women who elected to breast feed exclusively to those who elect to breast feed nonexclusively in terms of HIV transmission. Time-dependent Cox Proportional Hazards Models will be used. The statistical analysis will also involve an “intent-to-treat” analysis comparing those randomized to abrupt cessation of all breast feeding at 4 months with those randomized to continued breast feeding in terms of HIV-free survival using Kaplan-Meier life-table methods.

#### C- Study Procedures

The schedule of maternal and maternal/infant visits and sampling of blood and breast milk specimens is indicated in the table below.

Biological samples: Two 5ml tubes of blood will be obtained from the mother at or before 37 weeks of gestation for lymphocyte phenotyping, HIV RNA quantification and storage. One 5 ml tube of blood will be obtained from the child at 3 months for storage. Infant blood spots by heel stick will be collected at birth, 1 month and every 1 month to 6 months and every 3 months to 24 months for HIV DNA PCR assays. At 4.5-months post-partum, mothers will use a breast pump to obtain a sample of breast milk for determination of breast milk viral load and biological indicators of subclinical mastitis. Breast milk will also be collected at 1 week, 1 month, 4 months post-partum and every 3 months thereafter if the mother is still breastfeeding. Blood will be collected from mothers at 4 months, 12 months and 24 months post-partum for other measurements including CD4 counts and prolactin.

Counseling visits: From 38 weeks of gestation until delivery, women will return weekly to the clinic for prenatal lactation counseling. Lactation support will be provided immediately after birth to reinforce prenatal training and to promote the successful establishment of exclusive breast feeding. Counseling regarding infant feeding will be given at 1 week and at 1,2,3,4, 4.5, 5 and 6 months post-partum. Home visits interspersed between the clinic visits will occur to 4.5 months.

Clinical data collection: At enrollment, a short questionnaire regarding socio-demograpic information will be administered. Clinical neonatal and obstetric information will be collected at delivery. At each clinical visit (1 week, 1,2, 3, 4,4.5, 5, 6 months and every 3 months up to 24 months), weight and height of the child will be recorded and information regarding infant morbidity and feeding practices will be obtained according to standardized questions. The vital status of all children will be established through interview with the mother or home visits if required. Women who are breast feeding will receive a clinical breast assessment and history using standardized questions to document breast pain, engorgement, soreness, cracked nipples, and other symptoms.

Overview of the schedule of study visits in which biological samples will be collected,
counseling will be provided, and morbidity and other data collected.

|  | Prenatal weeks ––––––– | | |  | | Postnatal months –––––––––––––––––––––––––––––– | | | | | | | | | | | | |
| --- | --- | --- | --- | --- | --- | --- | --- | --- | --- | --- | --- | --- | --- | --- | --- | --- | --- | --- |
|  | <36 | <37 | <38 + weekly | Birth | 1w | 1 | 2 | 3 | 4 | 4.5 | 5 | 6 | 9 | 12 | 15 | 18 | 21 | 24 |
| Biological samples |  |  |  |  |  |  |  |  |  |  |  |  |  |  |  |  |  |  |
| Maternal blood draw | **X** |  |  |  |  |  |  |  | **X** |  |  |  |  | **X** |  |  |  | **X** |
| Breast milk sample |  |  |  |  | **X** | **X** |  |  | **X** | **X** |  | **X** | **if** | **if** | **if** | **if** | **if** | **if** |
| Infant blood spot |  |  |  | **X** | **X** | **X** | **X** | **X** | **X** | **X** | **X** | **X** | **X** | **X** | **X** | **X** | **X** | **X** |
| Infant blood draw |  |  |  |  |  |  |  | **X** |  |  |  |  |  |  |  |  |  |  |
| Counseling |  |  |  |  |  |  |  |  |  |  |  |  |  |  |  |  |  |  |
| Pre- & post-HIV test | **X** | **X** |  |  |  |  |  |  |  |  |  |  |  |  |  |  |  |  |
| Study consent | **X** | **X** |  |  |  |  |  |  |  |  |  |  |  |  |  |  |  |  |
| Infant feeding |  |  | **X** | **X** | **X** | **X** | **X** | **X** | **X** | **X** | **X** | **X** |  |  |  |  |  |  |
| Data collection |  |  |  |  |  |  |  |  |  |  |  |  |  |  |  |  |  |  |
| Baseline questionnaire |  | **X** |  |  |  |  |  |  |  |  |  |  |  |  |  |  |  |  |
| Neonatal / delivery |  |  |  | **X** |  |  |  |  |  |  |  |  |  |  |  |  |  |  |
| Infant morbidity |  |  |  |  | **X** | **X** | **X** | **X** | **X** | **X** | **X** | **X** | **X** | **X** | **X** | **X** | **X** | **X** |
| Feeding practices |  |  |  |  | **X** | **X** | **X** | **X** | **X** | **X** | **X** | **X** | **X** | **X** | **X** | **X** | **X** | **X** |

# D- Study Drugs

No specific study drugs will be given. However, to ensure that participation in this study is not coercive, two drug-based clinical protocols not routinely implemented in Zambia will be implemented for all women attending the study clinics regardless of whether or not they choose to enroll in this study. Trimethoprime-Sulfamethoxazole prophylaxis (150 mg TMP/m^2^/day with 750 mg SMX/m^2^/day orally as a single daily dose administered three times per week on consecutive days will be made available to all children who are born to mothers identified as HIV-infected. Thrice weekly PCP prophylaxis will be given from 4 weeks through 12 months of age. For infants who test HIV-positive, treatment will continue for the duration of the study.

The two-dose nevirapine regimen found in the Uganda HIVNET 012 protocol to reduce perinatal trans­mission by 50% will be offered as a service to all women and their infants at the two sites. A single oral dose of 200 mg (Boehringer Ingelheim Pharmaceuticals) will be given to the mother at the onset of labor, and a suspension of 2 mg/kg will be given to the neonate before discharge (or within 72 hours of birth).

**E- Medical Devices**

Not applicable

# F- Study questionnaires

A short questionnaire will be administered at enrollment. The questionnaire will cover basic socio-demographic information including: age, parity, amount of education, housing circumstances (e.g., availability of piped water inside dwelling, type of latrine available, available energy source for cooking, availability of a refrigerator, size of dwelling, and number of other adults and children per dwelling), previous child deaths, intended duration of exclusive breast feeding, and estimated gestational age at enrollment.

A neonatal form will be completed on the child shortly after delivery. This form will include date of birth, gender, birth weight, mode of delivery, and whether or not the two nevirapine doses were given as recommended.

Several standardized questions will be administered to the mother at each clinical visit. Collectively these will collect information on 1) maternal breastfeeding practices since the last visit, 2) medical history of the child since last visit, 3) maternal symptoms of mastitis and, in case of infant death, 4) verbal autopsy.

**G. Study Subjects and Recruitment of Subjects**

1200 HIV-seropositive, healthy pregnant women will be recruited at two primary health clinics in Lusaka, Zambia (George Clinic or Chawama Clinic). All women who present for prenatal services will be offered testing for HIV-antibody as part of prenatal care services. All HIV testing will be voluntary, with signed, informed consent and pre- and post-test counseling. Consent for HIV testing is separate from consent for participation in this study. Women will be counseled by HIV counselors trained in Zambia following local counseling guidelines. As part of the post-test counseling, all women who test HIV+ will receive information regarding the risks and benefits of breast feeding and asked to make an informed choice regarding infant feeding practice. All HIV+ women and their infants will be offered the two-dose nevirapine therapy regardless of whether they choose to enroll in this study or what feeding practice they intend. The majority of Zambian women initiate breast feeding, however, for those women who indicate that they wish to avoid all breast feeding, HIV counselors will connect them with community services to obtain infant formula. Infant formula will not be offered at the two study clinics to avoid possibility of inadvertent incentive/ endorsement of breast milk substitutes.

HIV+ women who indicate their decision to breast feed will be eligible for enrollment into the study. A study counselor will explain the study, including the randomization (abrupt cessation of all breast feeding at 4 months or continued breast feeding), and all study protocols (including participation in the exclusive breast feeding counseling program and all requirements for study samples and visits). Consent forms approved by all the participating institutions will be used to document consent.

Women will be re­cruited into the study if they express willingness to comply with their randomized assignment and agree to the requirements of the study protocol. All women who agree to participate in the study will be counseled to breast feed exclusively to 4 months. Half of these women (regardless of their actual feeding practice) will be randomized to a counseling program that will encourage abrupt cessation of breast feeding at 4 months and transition to full replacement feeding, and half will be randomized to a program a counseling program consistent with standard practice (gradual introduction of weaning foods after 4 months and continued breast feeding if desired).

Inclusion criteria:

- HIV+ pregnant women
- Informed choice regarding infant feeding practice is to breast feed
- Lives within the catchment area of the clinic
- Received HIV test results at least 2 weeks before their expected date of delivery
- Absence of significant presenting illness that requires hospitalization
- Agree to adhere to the requirements of study participation

Exclusion Criteria:

- Informed choice regarding infant feeding practice is to provide breast milk substitute
- Lives outside catchment area

Because the primary purpose of this study is to investigate the efficacy of an intervention to reduce the risk of HIV transmission through breast feeding in a low resource setting, it is necessary to recruit a population of pregnant women and follow them and their children.

#### H- Confidentiality of Study Data

Each study participant will be assigned a unique study number. All field study personnel with access to identifiable information will be required to sign a confidentiality agreement in order to guarantee the security of information pertaining to individual study patients. The study number will identify all forms pertaining to the results of interviews, the clinical examinations, and the laboratory tests. Computer data sets generated from such forms will omit identifiable data. Linkage of results and identifiable data will be done only when necessary (e.g., to schedule appointments and trace defaulters) by authorized study personnel. The file with the linked names and study numbers will be password-protected with very limited access. Statistical analyses of the study results will be presented in aggregate format in technical reports and in manuscripts submitted for publication in scientific journals. Such reports will not permit the identification of individual study subjects. Computer files containing all data collected in the study, stripped of all identifiable information, will be compiled into a final database.

**I- Location of the Study**

The study will be conducted at two primary care facilities (George and Chawama Clinics) located in Lusaka, Zambia, a resource poor country in south-central Africa which is burdened with one of the highest HIV/AIDS burden in the world.

The study will seek ethical clearance from all institutions participating in the study.

#### J- Risks and Benefits

The purpose of the study is to quantify the competing risks associated with different breast feeding practices (exclusive vs. nonexclusive) and long vs. short breast feeding. Since there are risks associated with breast feeding (HIV transmission) and risks associated with not breast feeding (non-HIV infectious disease mortality), the study aims to consider empirically the best option for women in very low resource settings.

A potential risk of the study is disclosure of HIV status. In order to support what may be a difficult practice i.e. to abruptly discontinue breast feeding at 4 months of age, women will be encouraged to disclose their HIV status to and enlist the help of the their spouses and extended family members. We recognize that, though in many settings disclosure of HIV infection status to one’s partner or family is desirable, in certain instances, it may result in untoward effects including possible violence. Individual counseling with women and with their partners (if requested by women) will be undertaken by trained counselors on-site who will attempt to facilitate these difficult processes. Shortened breast feeding may be easier to conceal than complete avoidance of all breast feeding.

Reduced mother-to-child transmission of HIV due to nevirapine therapy is expected and infected infants may benefit from bactrim prophylaxis. Women who participate in the study may benefit from lactation counseling and experience fewer complications such as sore nipples, breast infections, etc. Information regarding appropriate weaning foods and hygienic food preparation and the availability of fortified weaning foods may reduce the incidence of diarrheal disease and improve nutritional status of the infants. All study subjects will be monitored for potential adverse effects. All participants will be encouraged to contact the project coordinator, principal investigator, or other staff member to report any undesirable effects associated with the study. These reports will be brought to the attention of the medical director, and appropriate steps will be taken to resolve the problem, including reporting to the PI, withdrawal from the study, and/or medical treatment when appropriate.

If short term exclusive breast feeding proves to be a viable option to minimize these competing risks, this intervention will offer an effective, feasible, and potentially sustainable option to minimize postnatal mother-to-child transmission of HIV among HIV+ women living in very low resource settings.

**K- Alternative Therapies**

Not applicable

#### L- Compensation to Subjects

Participants will be compensated for travel and study participation for each study visit. The precise amount is determined by the local investigators. There are no costs to the study subjects.

#### M- Minors as Research Subjects

As this study involves the participation of minors.

# N- Radiation or Radioactive Substances

Not applicable

#### Lay Summary

**Short Exclusive Breast Feeding to Reduce Postnatal Transmission of HIV Through Breastmilk**

#### Study Purpose

Mothers who are infected with HIV can pass the infection to their babies by breast feeding. However, in poor countries, babies who are not breast fed are more likely to grow poorly and get sick from non-HIV diseases. The purpose of this study is to find out whether or not feeding only breast milk, called exclusive breast feeding, can reduce the chance that an HIV-infected mother will pass HIV to her baby through breast milk. We will learn if stopping breast feeding quickly after 4 months of exclusive breast feeding will reduce the number of babies who die because of HIV infection and non-HIV infectious diseases.

#### Study Design

The study is designed as a randomized experiment with specific non-experimental aims. The design has been selected to address the primary research objectives within practical and ethical constraints. Women presenting for prenatal care at two primary health clinics in Lusaka, Zambia, will be asked if they want to know their HIV status. All women who test HIV+ will be offered the two-dose nevirapine therapy (to reduce the chance that the mother will pass the HIV infection to the baby at birth). HIV+ women will also receive balanced information about the benefits and risks of infant feeding practices. Women who choose to breast feed will be invited to participate in the study. They will be encouraged to exclusively breast feed for the first four months after delivery. Half of the women will be randomized to stop breast feeding quickly and will be given locally-made weaning foods. The other half will be advised to continue to breast feed and gradually add other foods/liquids to the babies’ diets consistent with usual practice. Women and their children will be followed with regular study visits for two years to determine HIV status and under-2-year mortality. We will be able to answer whether it is better for babies to stop receiving breast feedings (lower chance of HIV infection, but higher chance of other sickness) or to continue to receive breast feedings (increased chance of HIV infection, lower chance of other sickness).

#### Study Subjects

1200 pregnant HIV-infected women will be recruited at two primary health clinics in Lusaka, Zambia. The participants are expected to range in age from 16 to 40 years. Half of the women will be assigned to abrupt cessation of breast feeding (intervention group) and the other half will be assigned to continued breast feeding (control group).

#### Recruitment Method

All women who enroll for prenatal services at George or Chawama Clinics will be offered pre-test counseling for HIV testing. Signed informed consent will be obtained for HIV testing. As part of the post-test counseling, women who test HIV+ will be counseled about the risks and benefits of breast feeding and asked to decide which feeding method they intend to use. Women who indicate that they will breast feed will be eligible for the study. Women who choose breast milk substitutes will receive assistance from the study counselors to link them with community services but infant milk formula will not be offered at the clinic.

**Study Procedures**

The study requires participation in the infant feeding counseling program which will involve weekly visits from 38 weeks until delivery, and monthly visits to 6 months. Blood will be drawn from the mother at enrollment, and at 4, 12, and 24 months after birth. Blood will be drawn from the infant at 3 months and heelstick blood spots will be collected at birth, 1 month and every month to 6 months and every 3 months up to 24 months. Breast milk will also be collected. Clinical data including socio-demographic and breast feeding behaviors will be collected at every clinical visit.

#### Issues

In wealthy countries, infant formula is available to reduce the chance that mothers pass HIV to their infants after birth. However, in poor countries, HIV-positive women cannot simply be advised to formula feed since infant formula is not safe and is usually not available. The purpose of this study is to test which is better -- long or short breastfeeding -- taking into account the competing risk of HIV versus non-HIV-related death and illness. A second aim of the study is to test a previous finding that exclusive breast-feeding (i.e. breast-milk only) conveys less risk of HIV than the more usual practice of mixed breast feeding. We already know that exclusive breast-feeding conveys much less risk of non-HIV-related death and illness. Being HIV+ may result in stigmatization and possible risk of violence in the community. Not breast feeding may expose women who want to keep their HIV status a secret. We will encourage, but not force, women to disclose their HIV status to their partners and family to enlist their support.
